# Right Iliac Ectopic Kidney With an Extrarenal Pelvis, Aberrant Upper-Pole Arterial Supply, and Multiple Renal Cysts

**DOI:** 10.7759/cureus.106074

**Published:** 2026-03-29

**Authors:** Chrysovalantis Mariorakis, George Triantafyllou, Ioannis Gogoulis, Konstantinos Natsis, Maria Piagkou

**Affiliations:** 1 Department of Paediatric Surgery, Hippokration General Hospital, Thessaloniki, GRC; 2 Department of Anatomy, School of Medicine, Faculty of Health Sciences, National and Kapodistrian University of Athens, Athens, GRC; 3 Department of Anatomy and Surgical Anatomy, School of Medicine, Faculty of Health Sciences, Aristotle University of Thessaloniki, Thessaloniki, GRC

**Keywords:** ectopic kidney, extrarenal pelvis, iliac kidney, pelvic kidney, variation

## Abstract

Renal ectopia results from an abnormal arrest of renal ascent during embryonic development and is often associated with malrotation, unusual vasculature, and anomalies of the collecting system. Although numerous ectopic kidneys are asymptomatic and discovered incidentally, their variant anatomy holds clinical significance as it may interfere with imaging interpretation and surgical procedures. We present a case of a right iliac ectopic kidney identified during the routine dissection of an 82-year-old male donor. The kidney was located in the right iliac fossa and demonstrated marked anterior malrotation, a large extrarenal pelvis, a solitary aberrant artery originating from the right common iliac bifurcation and entering the superior pole, venous drainage into the right common iliac vein, and multiple cortical and medullary cysts. The contralateral kidney was situated in its normal position and was grossly unremarkable. This combination of positional, vascular, collecting-system, and parenchymal variations exemplifies the complexity of renal ectopia and underscores the importance of meticulous anatomical identification. Understanding these variants is essential for radiologists, surgeons, and anatomists, as failure to recognize an ectopic kidney may result in diagnostic errors or iatrogenic injury during pelvic and vascular interventions.

## Introduction

Ectopic kidney results from the disruption of the normal ascent of the metanephric kidneys from the pelvis to the lumbar region during the fifth to ninth weeks of gestation. The pelvic kidney is the most common form, with a reported incidence ranging from approximately 1 in 500 to 1 in 1,100 births, although this varies across populations [1,2]. As many individuals remain asymptomatic, these kidneys are frequently detected incidentally during imaging, surgical procedures, or postmortem examinations [1,2].

In typical anatomy, the kidneys are located in the retroperitoneal space on either side of the vertebral column, typically extending from T12 to L3, with the right kidney positioned slightly lower due to the presence of the liver. During embryogenesis, the metanephric kidneys initially develop in the pelvic region and ascend cranially while undergoing medial rotation and sequential changes in vascular supply. Ectopic kidneys are commonly associated with abnormalities in rotation, vascular supply, and the collecting system. Malrotation often results in an anteriorly oriented hilum, while the ureter may be short or tortuous. The renal pelvis is frequently located outside the renal sinus. Vascular supply is highly variable and may arise from the iliac vessels or distal aorta, often involving multiple arteries, polar branches, and atypical venous drainage patterns [3-8].

From a clinical perspective, ectopic kidneys are important because they may mimic pelvic pathology, including tumors, lymphadenopathy, and adnexal masses [1,9,10]. Their anomalous vascular anatomy can complicate surgical procedures, particularly those involving the abdominal aorta or pelvic vessels, where inadvertent injury to variant arteries may result in renal ischemia [11]. In addition, malrotation and the presence of an extrarenal pelvis may predispose to urinary stasis, increasing the risk of hydronephrosis, infection, vesicoureteral reflux, and stone formation [4,12,13].

The present report describes a right iliac ectopic kidney identified during cadaveric dissection, with emphasis on its anatomical variations and their clinical relevance.

## Case presentation

During routine cadaveric dissection of the posterior abdominal wall in an 82-year-old male donor, the right kidney was not identified in its usual retroperitoneal position at the level of T12-L3. Further exploration of the abdominal and pelvic cavities revealed an ectopic kidney located in the right iliac fossa, positioned just superior to the pelvic brim and anterior to the psoas major muscle.

The kidney exhibited a globular configuration with loss of the typical reniform shape. It was markedly malrotated, with the hilum directed anteriorly rather than medially. An extrarenal pelvis was present and projected anteriorly into the peritoneal cavity. No posteriorly oriented renal sinus structures were identified.

The arterial supply was atypical. A single renal artery arose from the right common iliac artery immediately proximal to its bifurcation into the external and internal iliac arteries and entered the kidney at the upper pole rather than at the hilum. No renal artery originating from the abdominal aorta was observed. Shortly after entering the parenchyma, the artery divided into multiple segmental branches supplying the kidney.

Venous drainage occurred via a superficial renal vein that emerged anteriorly and drained into the right common iliac vein. No additional venous variations were identified.

The renal parenchyma demonstrated multiple cortical and medullary cysts of varying sizes, ranging from a few millimeters to more than 1 cm. The capsule was intact but appeared thinned overlying larger cysts. No calculi, hydronephrosis, or scarring were observed, although cortical thinning was evident.

The ureter followed an elongated course, passing anterior to the iliac vessels before descending toward the bladder. It was single, patent, and showed no evidence of duplication, kinking, or obstruction. The contralateral kidney was normally positioned, with typical orientation, vasculature, and ureter. No additional abnormalities were identified in the abdominal organs.

**Figure 1:**
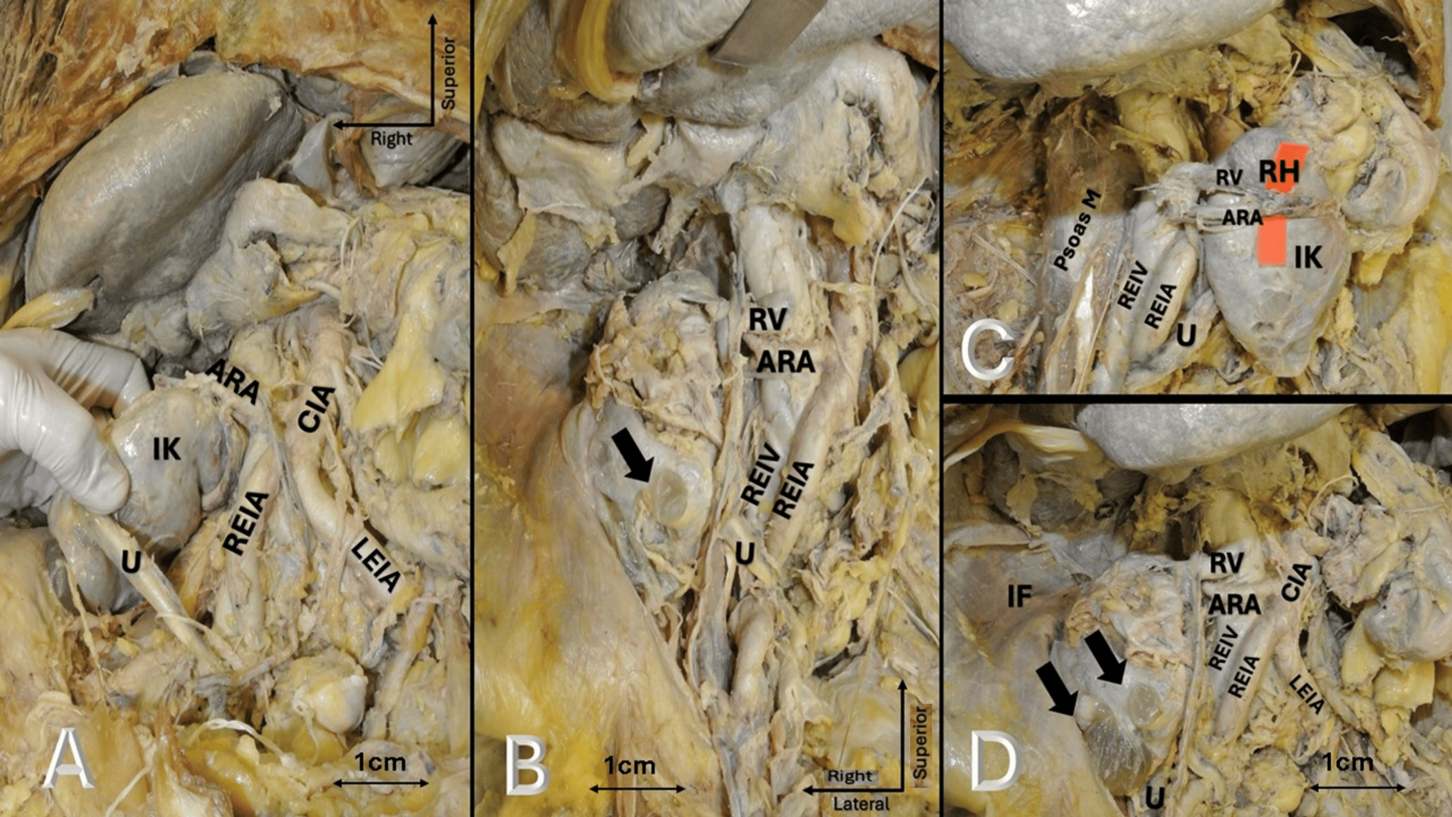
Right iliac ectopic kidney demonstrates positional, vascular, and parenchymal variations. (A) The iliac kidney (IK) is located in the right iliac fossa (IF), inferior to its typical lumbar position. The aberrant renal artery (ARA) arises from the common iliac artery (CIA). The ureter (U) descends inferiorly, and the right and left external iliac arteries (REIA and LEIA) are identified. (B) Closer view of the renal hilum showing the ARA and renal vein (RV). The ureter (U) is seen coursing inferiorly. The REIA and the right external iliac vein (REIV) are also visible. The arrow indicates cystic changes within the renal parenchyma. (C) The anteriorly oriented renal hilum (RH) demonstrates malrotation of the ectopic kidney (IK). RV and ARA are seen at the hilum. The U descends inferiorly, and the kidney lies anterior to the psoas major muscle. (D) The ectopic kidney is shown within the IF. The RV, ARA, and CIA are visible. The U is identified, and arrows indicate multiple cortical cysts on the renal surface. Orientation markers indicate anatomical directions. Scale bar = 1 cm

Overall, these findings are consistent with a right iliac ectopic kidney associated with anterior malrotation, a prominent extrarenal pelvis, a solitary aberrant artery entering at the upper pole, atypical venous drainage into the common iliac vein, and diffuse cystic changes (Figure 1).

## Discussion

Renal ascent takes place between the fifth and ninth weeks of gestation, during which the metanephric kidneys move from their initial pelvic position to the lumbar region. As they ascend, the kidneys also rotate medially and their blood supply changes, with early branches from the common iliac arteries gradually being replaced by vessels arising from higher levels of the abdominal aorta. When this process is disrupted, the kidney remains in a lower position, such as the pelvis or iliac fossa. In such cases, the level of the arterial supply often reflects the point at which ascent was arrested. In the present case, the renal artery arising from the right common iliac artery, just proximal to its bifurcation, suggests that ascent was interrupted at an early stage [1,4]. Malrotation is commonly associated with renal ectopia and results from incomplete rotation during ascent. Normally, the renal hilum turns medially; however, when rotation fails, it remains directed anteriorly. The anteriorly oriented hilum observed here, together with the forward projection of the extrarenal pelvis, is in keeping with incomplete rotation and supports a developmental origin for these findings [3-6].

Ectopic kidneys demonstrate considerable variation in vascular anatomy, rotation, and collecting system morphology. Most reports describe multiple renal arteries arising from the iliac vessels or distal aorta. In contrast, the present case showed a single aberrant artery arising from the proximal common iliac bifurcation and entering the kidney at the upper pole. This pattern is less frequently reported and differs from the more typical multiple arterial supply, with important implications for surgical planning [3,5,6].

An extrarenal pelvis is a recognized feature of ectopic kidneys and is often associated with impaired urinary drainage. In this case, the pelvis was not only extrarenal but also prominently anterior, likely reflecting the combined effects of malrotation and ectopia. Similar configurations have been associated with ureteropelvic junction obstruction and an increased risk of urinary stasis [4,7,12].

Ectopic kidneys may also pose diagnostic challenges because of their atypical location and morphology. Several reports have documented cases in which such kidneys were mistaken for pelvic masses or lymphadenopathy. The globular shape and iliac location observed in the present case could lead to similar diagnostic confusion if not recognized [1,8-10].

Although the contralateral kidney was normal, unilateral ectopic kidneys remain clinically relevant due to their abnormal vascular and ureteric anatomy, which may predispose to obstruction, infection, or injury.

From a surgical perspective, vascular configuration is particularly important. The presence of an aberrant artery arising from the iliac system, combined with venous drainage into the common iliac vein, increases the risk of inadvertent injury during pelvic or vascular procedures. This has been emphasized in both classical and contemporary surgical literature [11,14].

The diffuse cystic changes observed in this case add further complexity. While these cystic changes may be age-related, their presence in an ectopic kidney could also be associated with altered hemodynamics or impaired drainage; however, this cannot be definitively established in the present case. Similar findings have been reported in both cadaveric and clinical studies [15,16].

Advances in imaging, particularly multidetector computed tomographic urography, have improved the preoperative identification of vascular and ureteric variations in ectopic kidneys, facilitating safer surgical planning [7].

Although derived from a cadaveric specimen, the anatomical features observed in this case have direct clinical relevance. The aberrant arterial supply, particularly its entry at the upper pole, represents a potential source of vascular injury during pelvic or vascular procedures. The elongated ureter and its anterior course over the iliac vessels may predispose it to compression or trauma. The presence of an extrarenal pelvis raises the possibility of urinary stasis, increasing the risk of infection or stone formation. In addition, the diffuse cystic changes within the renal parenchyma may indicate long-standing functional compromise.

The unusual iliac location and altered morphology of the kidney further emphasize the importance of including an ectopic kidney in the differential diagnosis of pelvic masses. Failure to recognize this entity may result in misdiagnosis or inappropriate surgical intervention [1,8-10,17-20]. Careful radiological evaluation is therefore essential, particularly when unexpected findings are encountered.

This study is limited by its cadaveric nature, which precludes correlation with clinical history, functional assessment, or imaging findings. The absence of prior medical records or surgical history also limits the ability to exclude acquired causes of anatomical variation, although no evidence of surgical intervention or scarring was identified during dissection. Additionally, as a single case report, the findings cannot be generalized but instead contribute to the existing anatomical literature.

## Conclusions

This case describes a right iliac ectopic kidney with anterior malrotation, a prominent extrarenal pelvis, a solitary aberrant artery entering at the upper pole, atypical venous drainage into the common iliac vein, and diffuse cystic changes involving both cortex and medulla. The coexistence of a single upper-pole arterial supply from the common iliac artery, marked malrotation, and widespread cystic changes represents an uncommon anatomical combination. These findings are consistent with early arrest of renal ascent and persistence of embryonic vascular patterns, accompanied by structural and parenchymal alterations. Given the variability of ectopic kidneys, careful recognition of such features is important in both anatomical and clinical settings. Awareness of these variations may help avoid diagnostic confusion and reduce the risk of inadvertent injury during pelvic or vascular procedures.
